# Preclinical research progress in HER2-targeted small-molecule probes for breast cancer

**DOI:** 10.1007/s00117-024-01338-5

**Published:** 2024-07-22

**Authors:** Yefan Sun, Luoping Zhai, Le Ma, Wanchun Zhang

**Affiliations:** 1 Department of Medical Imaging, Shanxi Medical University, 030001 Taiyuan, China; 2 Third Hospital of Shanxi Medical University, Shanxi Bethune Hospital, Shanxi Academy of Medical Sciences Tongji Shanxi Hospital, 030032 Taiyuan, China

**Keywords:** Mammary cancer, Genes, HER2, Radiolabeled molecular probes, Molecular imaging, Prognosis, Mammarkarzinom, HER2-Gene, Radiomarkierte molekulare Sonden, Molekulare Bildgebung, Prognose

## Abstract

Breast cancer is a malignant tumor that has the highest morbidity and mortality in women worldwide. Human epidermal growth factor receptor 2 (HER2) is a key driver of breast cancer development. Therefore, accurate assessment of HER2 expression in cancer patients and timely initiation or termination of anti-HER2 treatment are crucial for the prognosis of breast cancer patients. The emergence of radiolabeled molecular probes targeting HER2 makes this assessment possible. This article describes different types of small-molecule probes that target HER2 and are used in current preclinical applications and summarizes their advantages and disadvantages.

## Abstract (de)

Brustkrebs ist ein maligner Tumor und weist die höchste Morbidität und Mortalität bei Frauen weltweit auf. Der humane epidermale Wachstumsfaktorrezeptor 2 (HER2) ist ein wesentlicher Treiber der Entstehung von Brustkrebs. Daher ist die genaue Untersuchung der HER2-Expression bei Brustkrebspatientinnen und die rechtzeitige Einleitung oder Beendigung einer Anti-HER2-Behandlung entscheidend für die Prognose von Brustkrebspatientinnen. Das Aufkommen radiomarkierter molekularer HER2-gerichteter Sonden ermöglicht diese Untersuchung. Im vorliegenden Beitrag werden die verschiedenen Typen niedermolekularer HER2-gerichteter Sonden, die aktuelle präklinisch eingesetzt werden, beschrieben und ihre Vor- und Nachteile zusammengefasst.

## Background

Breast cancer is the malignant tumor with the highest morbidity and mortality in women worldwide [1]. Compared with developed countries, the incidence of breast cancer in China is low, although it is increasing [2]. Human epidermal growth factor receptor 2 (HER2) is a key driver of breast cancer development [3] and is expressed at high levels in about 20–25% of women with breast cancer [4]. HER2 plays an important role in the regulation of cell proliferation, migration, and differentiation, which is the main reason for the high degree of malignancy, high recurrence rate, and poor prognosis associated with these tumors. Accurate and timely evaluation of HER2 expression in primary and metastatic lesions of breast cancer is therefore an important part of targeted therapy [5]. Overexpression of HER2 has the characteristics of both spatial and time heterogeneity. Traditional examination methods such as immunohistochemical staining (IHC) and fluorescence in situ hybridization (FISH) involve invasive procedures and therefore cannot be used widely or multiple times in patients [6]. As a consequence, in vivo real-time and dynamic monitoring of HER2 expression status cannot be achieved. The development of HER2-targeted nuclear medicine imaging technology makes it possible to monitor the expression of HER2 in the lesion in real time and in a noninvasive manner. Compared with single-photon emission computed tomography/computed tomography (SPECT/CT) and positron emission tomography/computed tomography (PET/CT), HER2-targeted nuclear medicine imaging provides better spatial resolution, registration efficiency, and accurate quantification of activity [7]. The ideal HER2-targeted molecular probe is the basis and key for achieving this goal.

## Development of HER2-targeted molecular probes

An ideal HER2-targeted molecular probe should have the characteristics of rapid tissue penetration, high affinity and specificity, appropriate blood clearance rate, high target-to-non-target ratio, low immunogenicity, easy preparation, and low production cost. HER2-targeted radiolabeled antibodies are limited in clinical application due to their immunogenicity, high molecular weight, low tissue penetration, long optimal imaging time (3–5 days), and complex preparation process. Although the antibody fragments derived from the complete antibody probe have a low molecular weight, immunogenicity, and optimal imaging time, the disadvantage of a poor display of the lesion site has become a major factor hindering their clinical application. In recent years, increasingly smaller molecular HER2-targeted probes have been developed, including radiolabeled affibodies, nanobodies, and peptides. These probes change the pharmacokinetics by connecting to other functional groups to obtain the best imaging performance. Compared with radiolabeled antibodies and antibody fragments, small-molecule HER2-targeted molecular probes have the characteristics of a very small structure, easy modification, strong tissue penetration, and rapid blood clearance, thereby providing great prospects for further development. This article focuses on the research progress of these three types of small-molecule probes.

## Radiolabeled affibodies

An affibody, also known as an “artificial antibody,” is a new type of scaffold protein based on non-immune protein affinity ligands derived from the immunoglobulin binding region B segment of Staphylococcal protein A [8, 9]. Compared with the antibody, the affibody has the following advantages: (1) simple structure, small molecular weight, high stability, easy preparation and storage [7], high temperature resistance [10, 11], and a tolerance to a wide range of acids and alkalis [10–13]; (2) high selectivity and affinity, low non-specific binding; and (3) strong tissue penetration, rapid concentration in the target tissue, and rapid clearance in the blood, which significantly reduces background uptake [14]. However, the disadvantages of poor image contrast caused by high background liver uptake and high production costs are still difficult problems that need to be solved in future research on affinity probes.

### ZHER_2:4_

ZHER_2:4_ is a first-generation HER2 affinity molecule. Due to its low affinity, it is difficult to use in targeted imaging [15]. Orlova et al. [16] used a secondary library design to perform site-directed mutagenesis of mature affinity sequences, and they finally obtained ten HER2 affinities (ZHER_2:473_, ZHER_2:382_, ZHER_2:470_, ZHER_2:487_, ZHER_2:489_、ZHER_2:477_, ZHER_2:492_, ZHER_2:475_, ZHER_2:342_, and ZHER_2:336_). In vitro studies have confirmed that ZHER_2:342_ has the highest affinity of the ten aforementioned compounds, reaching 22 pM, which is 2200 times higher than that of the initial ZHER_2:4_. The results of in vivo distribution studies showed that the uptake value of ^[125]^I‑labeled ZHER_2:342_ in tumors was double that of ZHER2:4. Therefore, based on their initial affinity, ZHER_2:4_ and ZHER_2:342_ produced by site-directed mutagenesis have the potential to become ideal HER2-targeted imaging agents.

In order to investigate the clinical application value of the probe ZHER_2:342_, which we also call “ABY-002,” Kramer-Marek et al. [17] connected *N*-[2-(4-Fluorobenzamido)ethyl]maleimide (FEBM) and thioserine (Cys) to ZHER_2:342,_ and compared the imaging performance of ^18^F(fluorine-18)-FBEM-Cys-ZHER_2:342_ and ^18^F‑fluoro-2-deoxyglucose(FDG) in a nude mouse model of lung metastasis in HER2-positive breast cancer. The results showed that ^18^F‑FBEM-Cys-ZHER_2:342_ was concentrated in HER2-positive metastases. The results of ^18^F‑FDG imaging also showed distribution in the surrounding inflammation and normal tissues, with no metastases detected. However, the complex preparation process, long time, low labeling rate, and high liver and intestinal uptake of the ^18^F‑labeled HER2 affinity probe have limited its clinical application [17]. Xu et al. [18] coupled the bifunctional chelating agents maleimide (MAL) and 1,4,7-triazacyclononylacetic acid (NOTA) with ZHER_2:342_ and then used Al^18^F(Aluminum fluoride-18) one-step labeling to obtain a new HER2 targeting probe: Al^18^F‑NOTA-MAL-MZHER_2:342_. In vitro competitive cell binding experiments showed that Al^18^F‑NOTA-MAL-MZHER_2:342_ had good targeting specificity. Due to the introduction of the hydrophilic groups, the liver uptake of ^18^F‑FBEM-Cys-ZHER_2:342_ and Al^18^F‑NOTA-MAL-MZHER_2:342_ was also reduced significantly, resulting in better contrast in the image. Furthermore, the total preparation time of Al^18^F‑NOTA-MAL-MZHER_2:342_ was approximately 30 min, while the non-attenuation correction yield at the end of the synthesis was 10%. Taken together, these results show that radiosynthesis of Al^18^F‑NOTA-MAL-MZHER_2:342_ is superior to that of ^18^F‑FBEM-Cys-ZHER_2:342_.

### ABY-025

ABY-025 is a second-generation affinity molecule. A ZHER_2:342_ site-directed mutagenesis of the amino acid sequence was used to obtain ZHER_2:981_, followed by ligation of the chelating agent 2,2′,2′′,2′′′-(1,4,7,10-Tetraazacyclododecane‑1,4,7,10-tetrayl)tetraaceticacid (DOTA) to form ZHER_2:981_ and linking it to cysteine. This generated second-generation affinity molecule had an increased melting point and also improved hydrophilicity and stability. Compared with the primary affinity of ZHER_2:342_ [19], ABY-025 has reduced liver uptake and avoids the situation in which metastases near the liver cannot be identified due to a high-contrast background. Sörensen et al. [20] conducted the first clinical trial of ABY-025 that involved performing a ^68^Ga (Gallium-68)-ABY-025 PET/CT imaging study using ^68^Ga-labeled ABY-025. The results confirmed the high specificity, affinity, and biosafety of the imaging agent, and changed the treatment of breast cancer in three of 16 patients.

## Nanobodies

To date, nanobodies are considered to be the smallest naturally derived antigen-binding fragments and are composed of the variable domain of the heavy chain part of camel immunoglobulin G [21]. Nanobodies have the characteristics of a low molecular weight (15 kDa), high affinity, low immunogenicity, strong tissue penetration, and easy binding to hidden antigens. They can be used clinically for both the treatment and diagnosis of tumors [22, 23]. At present, a variety of HER2-targeted nanobody probes have been developed, and research has confirmed the potential clinical application value of the nanobody 2Rs15d [24]. D’Huyvetter et al. [25] modified 2Rs15d with N‑succinimide 4‑guanidinomethyl-3-iodobenzoate (SGMIB), followed by radiolabeling with ^131^I. The subsequent SPECT imaging results showed that ^131^I‑SGMIB-2Rs15d had a faster clearance rate in the kidney than that of ^68^Ga-NOTA-2Rs15d, although the advantages of PET imaging were irreplaceable by SPECT. The biological half-life of HER2-targeted nanobodies matches the physical half-life of the nuclides ^68^Ga and ^18^F. In addition to the advantages of PET/CT imaging, the study of ^68^Ga and ^18^F‑labeled HER2 nanobodies has attracted increasingly more attention.

Xavier et al. [26] prepared the HER2-targeted probe [^18^F]-4-fluorobenzoate (FB)]–2Rs15d by coupling the most commonly used ^18^F‑labeled agent, ^18^F-(N-succinimidyl-4-fluorobenzoate), with the nanobody 2Rs15d and then carried out in vivo biodistribution and tumor imaging studies. The final results showed that ^18^F‑FB-2Rs15d was excreted mainly through the kidney with high image contrast, but that its radiochemical yield was only 5–15%. Another study by Xavier et al. [27] showed that the nanobodies 2Rs15dHis6 and 2Rs15d achieved rapid and efficient 68Ga labeling after being linked to the chelating agent NOTA used to generate the probes ^68^Ga-NOTA-2Rs15dHis6 and ^68^Ga-NOTA-2Rs15d, respectively. Biodistribution studies have shown that both of these probes can quickly and specifically accumulate in HER2-positive tumor sites and produce high-contrast PET/CT images. However, due to their high-contrast background in the liver, kidney, and intestine, the probes may not be suitable for evaluating HER2-positive primary breast lesions and did not compete with the anti-HER2 therapeutic antibodies trastuzumab and pertuzumab [28]. The probe is therefore expected to be used to monitor efficacy.

A study by Zhao et al. [29] used ^99m^Tc to label a single-domain antibody (sdAb) “called NM-02” with a radiochemical purity (RCP) of > 95%, pH = 7.0–7.5, and excellent in vitro stability. In their following study [30] of 30 newly diagnosed patients with breast cancer who had not received HER2-targeted therapy, they found a good relationship between ^99m^Tc-NM-02 SPECT/CT and pathological results. By contrast, no correlation was observed in the treated group. Compared with ^18^F‑FDG PET/CT, ^99m^Tc-NM-02 showed no non-specific uptake in inflamed tissues. In addition, the high production cost of the nanobodies has also limited their clinical transformation to a certain extent. However, interestingly, Altunay et al. [31] reported that ^99m^Tc-NM-02 SPECT/CT discriminated HER2 status in breast cancer, regardless of ongoing HER2-targeted antibody treatment. By using a plasma expander, the radiation dose to the critical organ (i.e., kidneys) appeared to be reduced by almost 50% without affecting uptake in the tumor sites, thereby effectively reducing renal uptake and making it possible for radioimmunotherapy of breast cancer. However, we consider that ongoing HER2-targeted antibody treatment will influence the uptake of ^99m^Tc-NM-02. In fact, because NM-02 is connected with the HER2 extracellular domain III [32], it does not compete with the anti-HER2 therapeutic antibodies trastuzumab and pertuzumab. Large-sample studies are required to determine why and how ongoing HER2-targeted antibody treatment influences the uptake of ^99m^Tc-NM-02.

## Peptides

In recent years, more and more peptides have been applied to the study of HER2-targeted radiomolecular imaging probes. These investigations have demonstrated the strong potential of radionuclide-labeled HER2-targeted peptides for HER2 imaging. Firstly, unlabeled small-molecule peptides have the advantages of a clear chemical structure, flexible modification space, controllable pharmacokinetic properties, non-immunogenicity, and easy synthesis. Secondly, radionuclide-labeled peptides have higher tissue permeability and a faster blood circulation time [33, 34]. However, in order to make HER2-targeted peptides an ideal nuclear medicine imaging agent, they usually need to be modified. HER2 is composed of an extracellular domain (ECD), a transmembrane domain, and an intracellular domain. The ECD is made up of four domains (I–IV; [35, 36]), and therefore these HER2 peptide probes are divided into the following two categories based on their different binding regions.

### HER2 extracellular domain II binding

#### KCCYSL peptide

The KCCYSL peptide is the most widely studied peptide probe at present and is composed of six amino acids that were discovered by phage display technology. A large number of SPECT imaging studies have confirmed that radiolabeled KCCYSL clearly reflects the expression of HER2 [37]. Kumar et al. [38] used ^64^Cu (copper-64) to label the KCCYSL peptide and studied the characteristics of three radiolabeled peptides with different modifications: ^64^Cu-tetraazacyclododecane-1,4,7-triacetic acid (DO3A)-GSG(Gly-Ser-Gly)-KCCYSL,^64^Cu-CB-1,4,8,11-tetraazabicyclo[6.6.2]hexadecane‑4,11-diac-etic acid (TE2A)-GSG-KCCYSL, and ^64^Cu-1,4,7-triazacyclononane‑1,4‑diacetic acid (NO2A)-GSG-KCCYSL. The 50.0% inhibitory concentration of these three peptides was 42.0 ± 10.2 nM, 31.79 nM, and 44.2 ± 6.6 nM, respectively. Furthermore, PET/CT imaging verified that these three radiolabeled peptide probes obtained clear images 2 h after injection. In addition, ^64^Cu-DO3A-GSG-KCCYSL had higher uptake and retention in the abdomen and liver, while the other two imaging agents were metabolized mainly by the kidney. Trastuzumab is also used in anti-HER2 therapy as it binds specifically to the extracellular ligand binding domain IV of the HER2 receptor, whereas KCCYSL binds to the extracellular domain II of HER2 [39]. Therefore, KCCYSL peptide-related targeted HER2 probes are expected to become one of the tools for monitoring changes in HER2 expression during trastuzumab targeted therapy.

#### H6F and H10F

The peptides H6F (YLFFVFER; [40]) and H10F (KLRLEWNR; [41]) were discovered using a high-efficiency peptide screening strategy based on an in situ single-bead sequencing microarray. Wang et al. [42] confirmed the HER2 targeting specificity of H6F and H10F; however, H6, an L‑type peptide, was shown to have poor stability in vivo. Therefore, on the basis of DU [43], the L‑type amino acid sequence was replaced with a D-type amino acid (DH6) and then reversed to a D-type peptide, RDH6, using reverse transcription. Polyethylene glycol (PEG) was then added to improve the water solubility of the peptide and its in vivo pharmacokinetics. The characteristics of high tumor uptake and less uptake in non-targeted organs, especially the liver, were finally confirmed by SPECT/CT imaging. In addition, H6F and H10F were shown to bind to the extracellular domain II of HER2, and have a function similar to that of the KCCYSL peptide for monitoring the efficacy of trastuzumab treatment.

### HER2 extracellular domain IV binding

Shadidi et al. [44] used a different phage display technique to discover another HER2-targeted specific peptide, LTVSPWY, while Ardakani et al. [45] used new chelating agents and linkers to form SSSLTVSPWY peptides and labeled these peptides with a positron nuclide ^68^Ga. A clearer image was obtained PET/CT imaging in the SKOV3 tumor model that better displayed the tumor tissue. However, compared with the KCCYSL peptide, the LTVSPWY peptide has the same binding site as trastuzumab in HER2, which may hinder trastuzumab-targeted HER2 treatment. The LTVSPWY peptide also does not have the ability to detect the therapeutic effect of trastuzumab and changes during treatment. However, as it binds to the same site as patozumab, it is expected that it will be used to monitor the efficacy of this treatment.

Peptide-targeted HER2 molecular probes have relatively low affinity, and therefore appropriate chemical modification is necessary. While improving their affinity, it is also necessary to maintain good pharmacokinetics as much as possible. Among the many peptide probes studied currently, the H6F probe has the best affinity, but it has a high hepatobiliary uptake. Therefore, RDH6 obtained on the basis of H6F has a clear molecular structure and is easy to modify. After PEG modification, RDH6 has improved stability in vivo, increased hydrophilicity, and reduced liver uptake. Furthermore, due to the addition of different chelating agents, RDH6 also has improved contrast in tumor imaging and can achieve the best imaging effect.

While investigating HER2-targeted molecular probes, we found that these probes have their own advantages and disadvantages. Some of the relevant pharmacokinetic data and advantages and disadvantages are summarized in Table 1. Although affinity-targeted and nanobody-targeted probes have high affinity and rapid blood clearance that result in better imaging contrast, their high uptake in the liver and kidney and the substantial production costs are the main problems hindering their development. By contrast, peptide molecular probes have better pharmacokinetic properties, non-immunogenicity, and easy preparation, making them a research hotspot for HER2-targeted probes. In this article, we mainly discuss the application of HER2-targeted molecular probes in the diagnosis of breast cancer. In their review, Altunay et al. [46] reported the in vivo use of radiolabeled antibodies enables the assessment of tumor heterogeneity, tumor accessibility, and the subsequent use of molecular targeted therapies. They also considered that the use of antibodies in molecular imaging was impaired by slow blood clearance and had limited potential to penetrate the tumor. Nanobodies therefore have properties that make them a preferable vehicle for molecular imaging as well as radioimmunotherapy.

**Table 1:** Pharmacokinetic data and advantages and disadvantages of HER2-targeted molecular probes

Probe types	Probes		Yield	IC50 or affinity constant	Main metabolic organs	Advantages	Disadvantages	Reference
Affibodies	^ABY-002^	^18^F‑FBEM-Cys-ZHER_2:342_	6.5 ± 2.2% (non-decay corrected)	15 nM	Kidney	Compared with 1^8^F‑FDG, there is no interference by surrounding inflammation and normal tissues	The preparation process is complex, the time is long, the labeling rate is low, and the liver and intestine uptake is high	Gabriela Kramer-Mark [17] Kramer-Marek G [47] Chopra A [48]
		Al^18^F‑NOTA-MAL-MZHER_2:342_	9.3 ± 1.5% (non-decay corrected)	116.71 ± 1.28 nM	Kidney	The total preparation time is about 30 min	The yield of non-decay correction was 10% at the end of synthesis	Xu Y [19] et al.
		Al^18^F‑NOTA-ZHER_2:342_	Non-decay-corrected maximum yield of 32.69%	–	Kidney	A radiochemical purity of more than 98%	–	Han J [49] et al.
	^68^Ga-ABY-025		40% (non-decay corrected)	–	Kidney	Compared with the primary affiliate ZHER_2:342_, it reduces liver uptake and avoids the inability to identify metastases near the liver due to high background	–	Sörensen J [21] Velikyan I [50]
Nanobodies	^18^F‑FB-2Rs15d		5–15% (decay corrected)	7.1 ± 0.4 nM	Kidney	High image contrast	Radiochemical yield is low and synthesis time is long	Xavier [27] et al.
	^68^Ga-NOTA-2Rs15dHis6 and ^68^Ga-NOTA-2Rs15d		^–^	4.64 ± 0.07 nM/3.91 ± 0.05 nM	Kidney	High image contrast	Liver, kidney, and intestines have a high-contrast background	Xavier [27]
Peptides	^64^Cu-KCCYSL		–	42 ± 10.2 nM/31 ± 7.9/ 44 ± 6.6 nM	Kidney	It is expected to become a tool for monitoring HER2 expression during trastuzumab targeted therapy	Higher uptake and retention in the abdomen and liver	KUMAR [38]
Etc	^99m^Tc-H6F and ^99m^Tc-RDH6		>95%	32.0 nM/10.3 nM	Kidney	Clear chemical structure, flexible modification space, controllable pharmacokinetic properties, no immunogenicity and easy synthesis	Poor stability in vivo, high hepatobiliary uptake	Wang [42], DU [43] et al.
	SSSLTVSPWY		–	–	–	It is expected to become a tool to monitor the expression of HER2 in the process of targeted therapy with pertuzumab	–	Ardakani [45] et al.

## Conclusion

As a broad tumor biomarker, HER2 plays an important role in tumorigenesis. Therefore, accurate assessment of HER2 expression in cancer patients and timely initiation or termination of anti-HER2 treatment are crucial for the prognosis of breast cancer patients. The value of these molecular probes targeting HER2 depends mainly on the purpose of their clinical application. For example, small-molecule probes can be used as an alternative to immunohistochemistry or fluorescent in situ hybridization techniques to diagnose the expression level of HER2 in tumors throughout the body. A complete antibody-targeted HER2 probe may be more suitable for evaluating the efficacy of targeted therapy for breast cancer. Although PET has better imaging quality, SPECT is more affordable. Therefore, it is helpful for timely monitoring of HER2 expression in patients to select the appropriate HER2-targeted probe for imaging according to different requirements such as diagnosis, efficacy evaluation, or treatment. This provides reliable real-time data for adjustment of treatment plans and evaluation of the curative effect.

## Declarations

For this article no studies with human participants or animals were performed by any of the authors. All studies mentioned were in accordance with the ethical standards indicated in each case.

The supplement containing this article is not sponsored by industry.
